# Supplementary material for: Heterogeneity of the GFP fitness landscape and data-driven protein design
Source: eLife. 2022 May 5;11:e75842. doi: 10.7554/eLife.75842 (PMC9119679; doi:10.7554/eLife.75842)
Supplement: Supplementary file 1. [file elife-75842-supp1.docx]

**Table S1. Selected statistics of genotypes at different divergence from five GFP sequences**

|  | Number of amino acid mutations | 1 | 2 | 3 | 4 | 5 | 6 | 7 |
| --- | --- | --- | --- | --- | --- | --- | --- | --- |
| amacGFP | Number of genotypes  Median fluorescence  Fraction wildtype-like*  Fraction dark**  Total possible genotypes  Fraction sampled*** | 1214  3.94  63.7%  9.0%  4760  25.6% (84%) | 10439  3.9  44.7%  11.7%  ~107  ~0.1% | 6389  3.85  31.2%  19.6%  ~1.5 · 10^10^  ~10^-7^ | 3077  3.75  21.6%  27.9%  ~1.7 · 10^13^  ~10^-10^ | 1269  3.44  15.2%  38.4%  ~1.5 · 10^16^  ~10^-13^ | 511  2.82  8.6%  55.0%  ~1.1 · 10^19^  ~10^-17^ | 188  2.74  8.5%  63.3%  ~7 · 10^21^  ~10^-20^ |
| amacGFP:V12L | Number of genotypes  Median fluorescence  Fraction wildtype-like*  Fraction dark**  Total possible genotypes  Fraction sampled*** | 1068  3.93  65.0%  5.8%  4760  22.4% (74%) | 5621  3.89  46.3%  10.7%  ~107  ~0.06% | 3010  3.85  33.0%  16.8%  ~1.5 · 10^10^  ~10^-7^ | 1313  3.78  24.1%  27.3%  ~1.7 · 10^13^  ~10^-10^ | 534  3.46  16.7%  42.1%  ~1.5 · 10^16^  ~10^-14^ | 207  2.83  9.7%  52.3%  ~1.1 · 10^19^  ~10^-17^ | 84  2.72  4.8%  67.9%  ~7 · 10^21^  ~10^-20^ |
| cgreGFP | Number of genotypes  Median fluorescence  Fraction WT-like*  Fraction dark**  Total possible genotypes  Fraction sampled*** | 1188  4.43  44.4%  14.5%  4700  25.3% (84%) | 10347  4.36  24.3%  23.7%  ~9.9 · 106  ~0.1% | 6567  3.62  11.4%  45.2%  ~1.4 · 10^10^  ~10^-7^ | 3666  2.79  5.7%  65.5%  ~1.6 · 10^13^  ~10^-10^ | 1959  2.77  2.0%  81.4%  ~1.4 · 10^16^  ~10^-13^ | 1061  2.77  0.9%  87.6%  ~10^19^  ~10^-16^ | 546  2.77  0.2%  92.3%  ~6.4 · 10^21^  ~10^-20^ |
| ppluGFP2 | Number of genotypes  Median fluorescence  Fraction wildtype-like*  Fraction dark**  Total possible genotypes  Fraction sampled*** | 1163  4.2  66.1%  4.4%  4440  26.2% (87%) | 16134  4.16  43.8%  10.2%  ~8.9 · 106  ~0.18% | 8920  4.1  29.7%  18.7%  ~1.2 · 10^10^  ~10^-7^ | 3710  3.96  19.6%  29.8%  ~1.2 · 10^13^  ~10^-10^ | 1370  3.67  16.0%  38.8%  ~1.1 · 10^16^  ~10^-13^ | 456  3.32  17.1%  44.5%  ~7.3 · 10^18^  ~10^-17^ | 186  2.86  11.3%  60.2%  ~4.3 · 10^21^  ~10^-20^ |
| avGFP | Number of genotypes  Median fluorescence  Fraction wildtype-like*  Fraction dark**  Total possible genotypes  Fraction sampled*** | 1114  3.64  68.9%  9.4%  4760  23.4% (90%) | 13010  3.59  55.5%  12.4%  ~107  ~0.13% | 12683  3.49  39.1%  27.0%  ~1.5 · 10^10^  ~10^-7^ | 9759  3.14  23.3%  47.9%  ~1.7 · 10^13^  ~10^-10^ | 7215  1.53  13.1%  68.4%  ~1.5 · 10^16^  ~10^-13^ | 4643  1.43  6.6%  83.0%  ~1.1 · 10^19^  ~10^-16^ | 2783  1.36  2.3%  92.0%  ~7 · 10^21^  ~10^-19^ |

*Fraction wildtype-like refers to the fraction of genotypes displaying fluorescence levels within two standard deviations of the wildtype, or brighter.

**Fraction dark refers to the fraction of fully non-functional genotypes (i.e. with fluorescence values falling within the darkest FACS gate). Remaining genotypes not accounted for in these two categories displayed a range of intermediate fluorescence levels.

***The number of possible protein sequences of length L with N mutations, starting from a wildtype sequence with 19 possible amino acid substitutions at each site, can be calculated as $\frac{L}{N}\cdot{19}^{N}$. At N>1, it is not experimentally feasible to sample more than a tiny fraction of the theoretical space. For N=1, the percentage of protein genotypes requiring only a single nucleotide mutation are also shown, in parentheses.
