# Supplementary material for: Heterogeneity of the GFP fitness landscape and data-driven protein design
Source: eLife. 2022 May 5;11:e75842. doi: 10.7554/eLife.75842 (PMC9119679; doi:10.7554/eLife.75842)
Supplement: Supplementary file 2. [file elife-75842-supp2.docx]

**Table S2.** Data Collection and Refinement Statistics

|  | **amacGFP (PDB ID 7LG4)** |
| --- | --- |
| Wavelength (Å) | 1.3418 |
| Space group | P1 2_1_ 1 |
| Unit cell dimensions | a = 34.089,  b = 47.488,  c = 69.294,  𝛼 = 90°,  𝛽 = 102.01°,  𝛾 = 90° |
| Resolution range (Å) | 22.59 - 1.81 (1.91 - 1.81) |
| Total no. of reflections | 151,191 |
| Unique reflections | 19,894 (2,932) |
| Completeness (%) | 99.8 (99.5) |
| Multiplicity | 7.59 (4.69) |
| Mean I / σ(I) | 9.36 (1.93) |
| CC_1/2_ | 0.996 (0.624) |
| **Refinement Statistics** | |
| Rwork/Rfree(%) | 18.17/22.75 |
| Average B factor (Å^2^) | 20.14 |
| Total no. of atoms | 2,051 |
| Protein atoms | 1,863 |
| Water molecules | 134 |
| Protein residues | 228 |
| Bond angles (°) | 1.91 |
| Bond length (Å) | 0.014 |
| Ramachandran: favored/allowed (%) | 98.65/1.35 |
| Clashscore | 5.77 |
| Numbers in parentheses are for the highest-resolution shell. | |
